# ADP-Ribosylation Factor-Like 2 (ARL2) regulates cilia stability and development of outer segments in rod photoreceptor neurons

**DOI:** 10.1038/s41598-018-35395-3

**Published:** 2018-11-16

**Authors:** Zachary C. Wright, Yuriy Loskutov, Daniel Murphy, Peter Stoilov, Elena Pugacheva, Andrew F. X. Goldberg, Visvanathan Ramamurthy

**Affiliations:** 1 Departments of Ophthalmology, West Virginia University, Morgantown, West Virginia 26506 USA; 2 Departments of Biochemistry, West Virginia University, Morgantown, West Virginia 26506 USA; 3 Center for Neuroscience, Robert C. Byrd Health Sciences Center, West Virginia University, Morgantown, West Virginia 26506 USA; 4 Eye Research Institute, Oakland University, Rochester, Michigan 48309 USA

## Abstract

Photoreceptor cells are specialized neurons with a sensory cilium carrying an elaborate membrane structure, the outer segment (OS). Inherited mutations in genes involved in ciliogenesis frequently result in OS malformation and blindness. ADP-ribosylation factor-like 2 (ARL2) has recently been implicated in OS formation through its association with Binder of ARL2 (BART or ARL2BP), a protein linked to inherited blinding disease. To test the role of ARL2 in vision we created a transgenic mouse model expressing a tagged-dominant active form of human ARL2 (ARL2-Q70L) under a rod-specific promoter. Transgenic ARL2-Q70L animals exhibit reduced photoreceptor cell function as early as post-natal day 16 and progressive rod degeneration. We attribute loss of photoreceptor function to the defective OS morphogenesis in the ARL2-Q70L transgenic model. ARL2-Q70L expression results in shortened inner and outer segments, shortened and mislocalized axonemes and cytoplasmic accumulation of rhodopsin. In conclusion, we show that ARL2-Q70L is crucial for photoreceptor neuron sensory cilium development. Future research will expand upon our hypothesis that ARL2-Q70L mutant interferes with microtubule maintenance and tubulin regulation resulting in impaired growth of the axoneme and elaboration of the photoreceptor outer segment.

## Introduction

The outer segment (OS) of photoreceptor neurons is a non-motile cilium that is specialized in light perception^1^. Defective OS formation affects vision and is often a consequence of impaired ciliogenesis in ciliopathic diseases^2,3^.

To gain insight into the development of the OS and related diseases, it is crucial to identify the proteins and events involved in this process. A number of small GTPases, which act as molecular switches, are believed to play a role in regulating protein-protein interactions throughout the process of ciliogenesis and OS formation^1,4–6^. These proteins are likely involved in the temporal and spatial regulation of ciliary proteins and add a level of complexity to photoreceptor morphogenesis. Specifically, the ADP-ribosylation factor-like 2 (ARL2) small GTPase was implicated in the process of OS formation in photoreceptor cells through its interaction of the Retinitis Pigmentosa (RP)-linked gene, ARL2 Binding Protein (ARL2BP)^7^. Importantly, ARL2 selectively interacts with ARL2BP in the GTP-bound active state^8^.

Despite the connection to OS formation through ARL2BP, there is little direct evidence for the role of ARL2 in photoreceptor neurons as well as *in vivo* function of this protein in general. Studies of ARL2 and ARL3, a close ARL2 homolog, suggest that they may have overlapping functions. Specifically, they are thought to regulate trafficking of prenylated proteins through their interaction with prenyl binding protein δ (PrBPδ)^9,10^. This function of ARL3 has been shown *in vivo* by multiple animal models, including dominant active transgenic ARL3-Q71L and conditional ARL3 knockout mice^11,12^. However, a role for ARL2 in prenylated protein trafficking has yet to be tested *in vivo*.

ARL2 has been implicated in a number of cellular processes^9,13–17^. ARL2 is thought to play a role in microtubule formation and regulation of the soluble tubulin pool^14,16,18–20^. A number of studies have shown that expression of dominant active ARL2 causes microtubule destruction by preventing polymerization^14,16,20^. More recent studies propose that active ARL2-GTP may interact with tubulin binding cofactors D and E (i.e. TBCD and TBCE) to regulate the state of tubulin dimerization and therefore the pool of free polymerizable tubulin^19^. In agreement with the proposed role in microtubule formation, knockdown of ARL2 or its binding partner ARL2BP results in shortening of cilia in ARPE-19 cell lines^7^. In addition, ARL2 is localized at the centrosome in cell lines where it is proposed to act as a microtubule regulator in an ARL2BP-independent manner^14^. These data suggest that ARL2 may play a role in OS formation by regulating tubulin or ARL2BP at photoreceptor cilium.

The purpose of this study was to gain insight into the role of ARL2 in rod photoreceptor cells by expression of the dominant active mutant ARL2-Q70L. Here we show for the first time that proper regulation of ARL2 is necessary for rod cilia regulation and OS formation.

## Results

### Endogenous *Arl2* Expression Profile and Dominant Active Mutant Transgenic Model Generation

**Figure 1:**
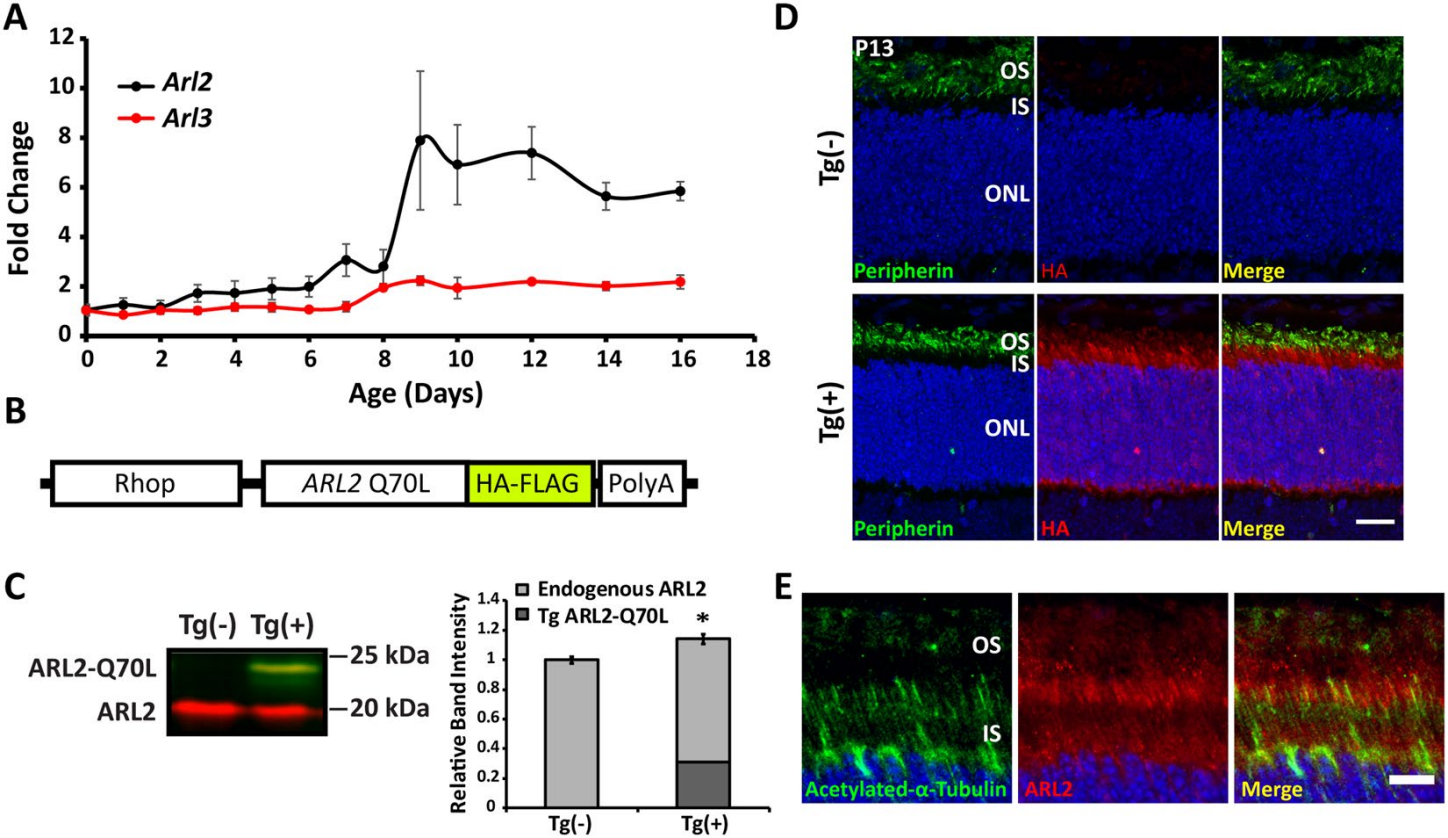
Expression of *Arl2* and Creation of Dominant Active Mutant Transgenic Model. This figure illustrates the developmental mRNA expression of *Arl2* and *Arl3* in mouse retina as well as the expression of dominant active ARL2 in our transgenic animal model. (**A**) *Arl2* and *Arl3* mRNA expression was determined by quantitative PCR of mouse retinal tissues from P0 to P16. Expression levels are displayed as fold change relative to the expression observed at P0 (n = 5). (**B**) This scheme illustrates the construct used for transgenic mouse generation. Human *Arl2* Q70L (ARL2 Q70L; glutamine to leucine dominant active mutant) expression is driven by a 4.4 Kb rhodopsin promoter (Rhop). The ARL2 Q70L protein is also tagged at the C-terminus with hemagglutinin (HA) and FLAG epitope. (**C**) Western blot analysis of endogenous versus transgenic ARL2 protein levels in P13 retinal tissue samples by staining with ARL2 (red) and HA (green) directed antibodies. Refer to bar graph for quantitation of total ARL2 (endogenous + transgenic ARL2-Q70L) expression levels relative to total ARL2 protein expression in controls using the ARL2 directed antibody (red) (n = 4; p = 0.012; * = p < 0.05). (**D**) Immunofluorescence analysis of retinal Tg(−) and Tg(+) cross sections from P13 animals. The localization of transgenic ARL2 was identified by incubating with HA directed antibody (red) and the OS were identified by staining with peripherin-2 (green) (Scale Bar = 20 μm). (**E**) Localization of endogenous ARL2 (using ARL2-directed antibody – red) and the ciliary marker acetylated tubulin (green) in P13 Tg(−) cross sections (Scale bar = 10 μm).

To understand the role of ARL2 in mouse photoreceptor cells, it is important to know the temporal dynamics of *Arl2* gene expression. We examined the developmental retinal mRNA expression profile of endogenous *Arl2* and *Arl3* in mice (Fig. 1A). We quantified by real time RT-PCR the message levels of *Arl2* and *Arl3* from post-natal day 0 (P0) through P16. As shown in the figure, *Arl2* expression remains low until a 4-fold spike in message levels between ages P6 and P9. This “switch” in expression suggests a functional need for ARL2 corresponding to elaboration of the OS, which begins at postnatal day 8. *Arl3* expression was also elevated between P6 and P9, but the change was moderate in comparison to *Arl2*.

To determine the role of ARL2 in photoreceptor function, we generated a transgenic animal expressing dominant active human ARL2-Q70L in rod photoreceptor cells (Fig. 1B). Expression of ARL2-Q70L in the transgene is under the control of a rhodopsin promoter (Rhop) that initiates the expression of transgenes as early as post-natal day 4 (P4) in rod photoreceptor cells of the retina^21^. This approach is similar to our recently published work where we expressed ARL3 in rod photoreceptor cells^11^. Transgenic founders were crossed with 129/SV-E wild-type mice for a few generations to ensure consistent transgene inheritance and expression prior to analysis. Over 8 transgenic founder lines were assessed for homogeneity of transgene expression and the lines utilized herein displayed the most homogeneous expression in photoreceptor cells. Transgenic negative littermates of ARL2-Q70L mice were used as control animals for comparison unless stated otherwise.

We generated an affinity purified rabbit antibody against ARL2. This antibody specifically recognized ARL2 and not its homologue ARL3 expressed in Human Embryonic Kidney (HEK293) cells (data not shown). In retina from transgenic animals at P13 using an ARL2-directed antibody (red), we find that ARL2-Q70L (~23 kDa) was expressed at 40% the level of endogenous ARL2 (~20 kDa) protein (Fig. 1C; n = 4; p < 0.001) while the total ARL2 (endogenous ARL2 + transgenic ARL2-Q70L) was ~14% greater than the total ARL2 protein in controls (Fig. 1C; n = 4; p = 0.012). Additionally, an anti-HA antibody (green) was used to confirm the identity of the tagged ARL2-Q70L, which overlaps with the ARL2 directed antibody fluorescence (red) at ~23 kDa (Fig. 1C).

The expression of transgenic ARL2-Q70L at P13 was confirmed by indirect immunofluorescence with anti-HA antibody (Fig. 1D). ARL2-Q70L was found in photoreceptor cells. Unlike transgenic and endogenous ARL3 localization examined in our previous study^11^, we observed intense transgenic protein fluorescence in the photoreceptor cell inner segment, ONL, and outer plexiform layer and only minimal overlap with the OS marker peripherin in the OS (Fig. 1D,E). Endogenous ARL2 fluorescence was observed throughout the IS and the OS with greater intensity in the IS-OS boundary (Fig. 1D,E).

Overall, these data demonstrate a switch in endogenous *Arl2* expression between P6 and P9, coinciding with the early stages of photoreceptor OS elaboration^22^, and that transgenic ARL2 is localized throughout photoreceptor cells with greatest abundance in the IS, ONL, and outer plexiform layer.

### Decreased photoreceptor function in ARL2-Q70L mice

**Figure 2:**
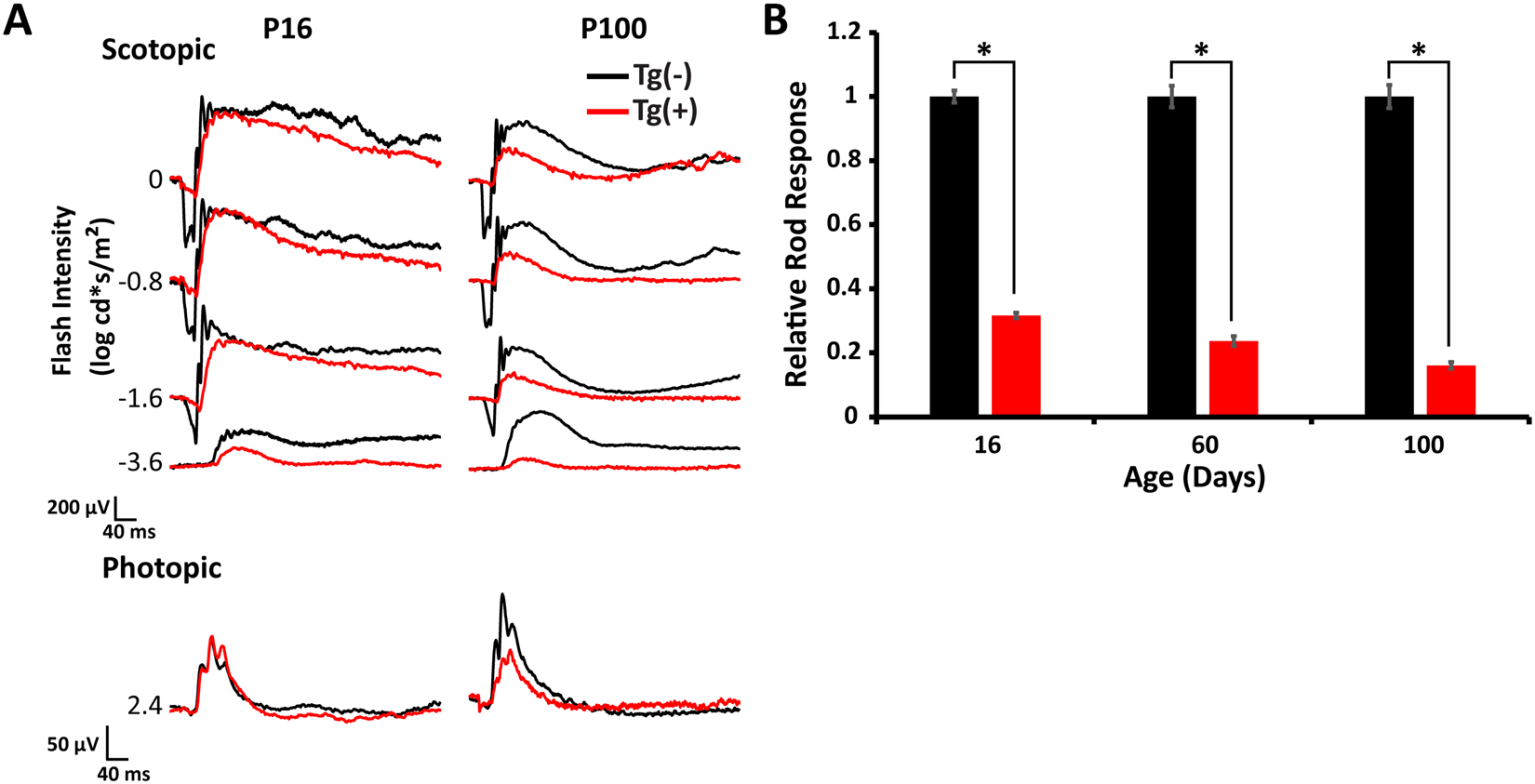
Reduced photoreceptor response in ARL2-Q70L mice. (**A**) Representative scotopic (rod) and photopic (cone) electroretinograms (ERGs) comparing Tg(−) and Tg(+) animals at P16 and P100 across multiple light intensities. (**B**) Graph showing the scotopic a-wave amplitude measured at the light intensity of −0.8 log (cd*s/m^2^) at different ages. n = 5; *p < 0.01.

We performed electroretinography (ERG) starting from P16 to determine the impact of expression of dominant active ARL2 on photoreceptor cell function. Transgenic ARL2-Q70L animals exhibited a major ~70% reduction in scotopic a-wave at P16 compared to transgenic littermate controls which showed a robust a-wave and b-wave (Fig. 2A; Tg(−) = 379.8 ± 7.2 μV and Tg(+) = 120.0 ± 3.3 μV, n = 5, p < 0.0001). As expected, cone photoreceptor response remained unaffected at this time point as transgenic negative and transgenic positive photopic b-waves were comparable (Fig. 2A; Tg(−) = 123.18 ± 6.4 μV and Tg(+) = 138.2 ± 1.9 μV; n = 5, p = 0.17). Rod photoresponse progressively declined in transgenic ARL2-Q70L animals with an 85% reduction in a-wave by P100 compared to littermate controls (Fig. 2A,B; Tg(−) = 379.3 ± 19.6 μV and Tg(+) = 60.7 ± 9.7 μV, n = 5, p < 0.0001). Finally, only a slight difference in rod sensitivity was noted with scotopic a-wave half-saturating light intensities of 0.010 ± 0.0019 cd*s/m^2^ in Tg(+) animals versus 0.014 ± 0.0018 cd*s/m^2^ in littermate controls and calculated maximum a-wave amplitudes of 225.2 ± 6.6 μV (Tg(+))and 499.7 ± 10.6 μV (Tg(−)). Taken together, these results illustrate that expression of ARL2-Q70L reduced rod photoreceptor function.

### Rod photoreceptor cells degenerate in animals expressing ARL2-Q70L

**Figure 3:**
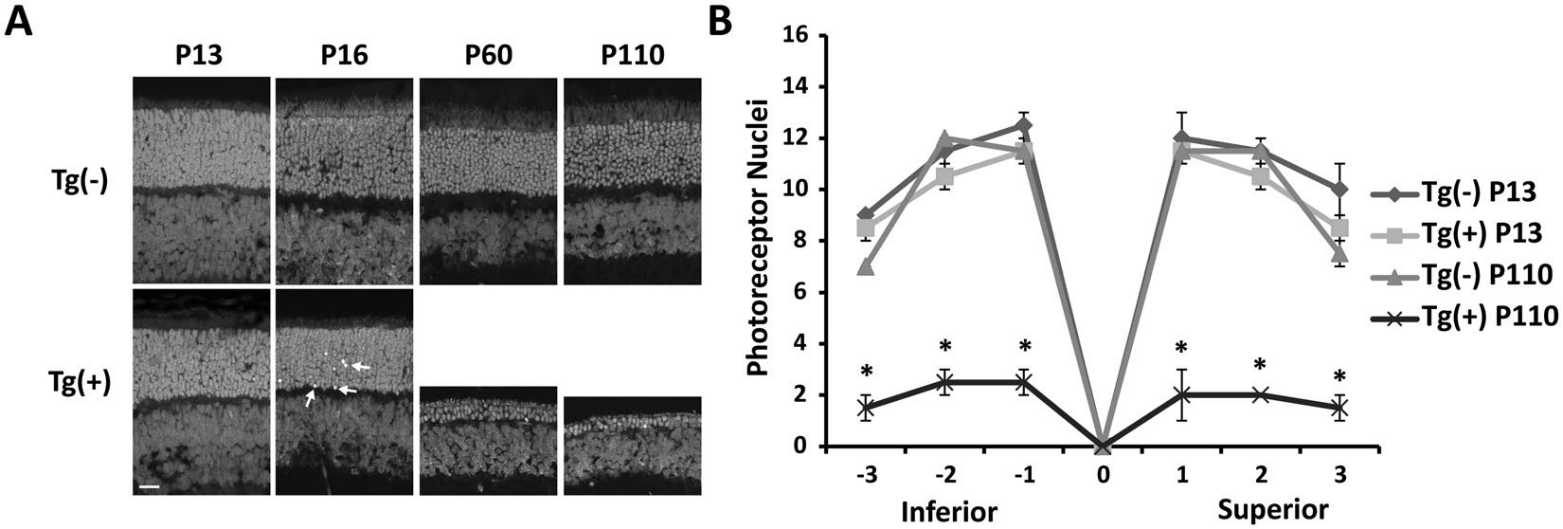
Degeneration of Rod photoreceptor cells in animals expressing ARL2-Q70L. (**A**) Retinal sections of Tg(−) and Tg(+) littermates stained with propidium iodide to demonstrate the ONL integrity at different ages (P13, P60, and P110). (**B**) Quantification of the ONL length (number of nuclei) between Tg(−) and Tg(+) littermates at different locations within the retina from the inferior (−3) to superior (3) portion at P13 and P110. n = 4; *p < 0.01 (Scale Bar = 20 μm).

We observed a gradual decline in photoreceptor function as the animals aged (Fig. 2B). To determine if the reduction in photoreceptor function is due to photoreceptor cell death, we examined the outer nuclear layer (ONL) length from P13 to P110. At P13, there was no difference in the size of the ONL (Fig. 3A,B; Tg(−) = 11.0 layers versus Tg(+) = 10.2 layers; n = 4, p = 0.26), however, there was a noticeable increase in the number of apoptotic nuclei observed with propidium iodide. At P16 apoptotic nuclei were more frequent with a minor decrease in the ONL length. (Fig. 3A, arrows indicate apoptotic nuclei). By P60 there was a major loss of photoreceptor cells and by P110 the ONL was nearly gone with only 1–3 nuclear layers remaining (Fig. 3A,B; P110: Tg(−) = 10.2 layers and Tg(+) = 2.0 layers; n = 4, p < 0.01 at all locations). These data suggest that defects during the development of rod photoreceptor cells results in progressive degeneration.

### Reduction of photoreceptor OS proteins ARL2-Q70L animals

**Figure 4:**
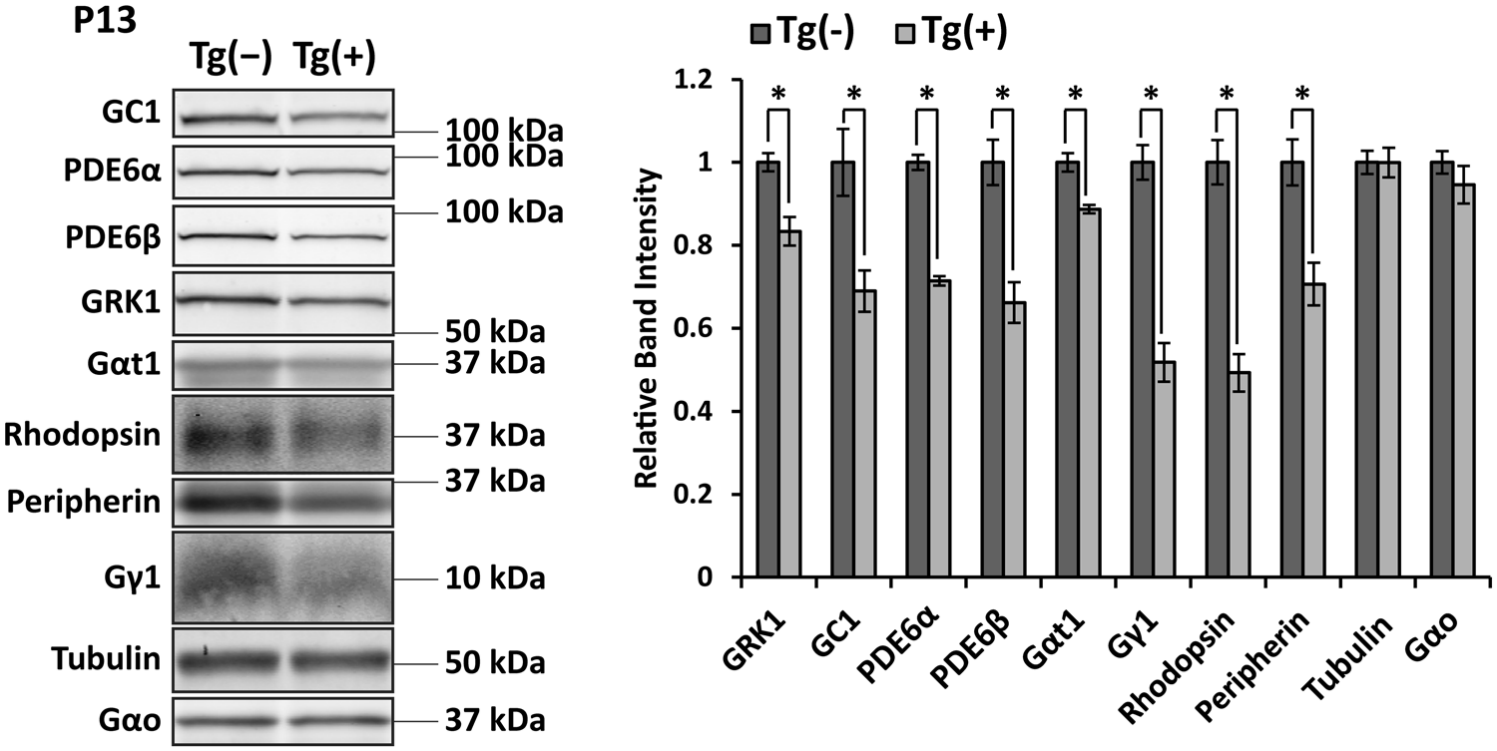
Reduced levels of photoreceptor OS proteins in ARL2-Q70L animals. Representative immunoblots showing expression of proteins from retinal lysates of Tg(−) and Tg(+) littermates at P13. Quantification of these protein levels is shown in the bar graph on the right. All samples were normalized to G_o_α, a protein expressed in the bipolar cells. n = 4, *p < 0.05.

The reduction in photoresponse of ARL2-Q70L animals at P16 does not correlate to the minor loss of cells at that age. This suggests a defect in the phototransduction cascade or a reduction in levels of phototransduction proteins. Previous *in vitro* studies have implicated ARL2 as a regulatory protein for prenylated cargo through its interaction with PrBPδ, similar to its homolog ARL3^9,10^. Therefore, we investigated the levels of prenylated phototransduction proteins along with other non-prenylated proteins in ARL2-Q70L expressing animals prior to degeneration (Fig. 4). The prenylated proteins phosphodiesterase 6 α (PDE6α) and β (PDE6β) subunits were significantly reduced by ~30% at P13 compared to transgenic negative littermates (PDE6α: P13 ≈ −30% p < 0.001; PDE6β: P13 ≈ −34% p < 0.01; n = 4). Similarly, farnesylated G-protein coupled receptor kinase 1 (GRK1), rod transducin γ subunit (Gγ1), and myristoylated rod transducin α (Gαt1) were all significantly reduced at P13 (GRK1: P13 ≈ −17% p < 0.01; Gγ1: P13 ≈ −50% p < 0.01; Gαt1: P13 ≈ −11% p < 0.01; n = 4). However, to our surprise, the non-prenylated OS proteins guanylate cyclase 1 (GC1), rhodopsin, and peripherin-2/rds were also significantly reduced by approximately the same magnitude as the prenylated proteins (GC1: P13 ≈ −31% p < 0.05; rhodopsin: P13 ≈ −51% p < 0.001; peripherin-2/rds: P13 ≈ −30% p < 0.05; n = . 4). A similar pattern was seen in P16 retinal samples as well. These proteins were all normalized to G_o_α, which is expressed in the bipolar cells and was unchanged between transgenic and littermate controls (P13: p = 0.33; n = 4). Additionally, tubulin was unchanged between transgenic animals and controls (P13: p = 0.99; n = 4). These results are in contrast to mouse models disrupting the ARL2 homolog, ARL3, which result in specific loss of prenylated proteins prior to cell death^11,12^. The broad decrease in photoreceptor OS proteins suggests that ARL2 is not acting specifically through PrBPδ to disrupt prenylated proteins.

### Mislocalization of Rhodopsin in ARL2-Q70L animals

**Figure 5:**
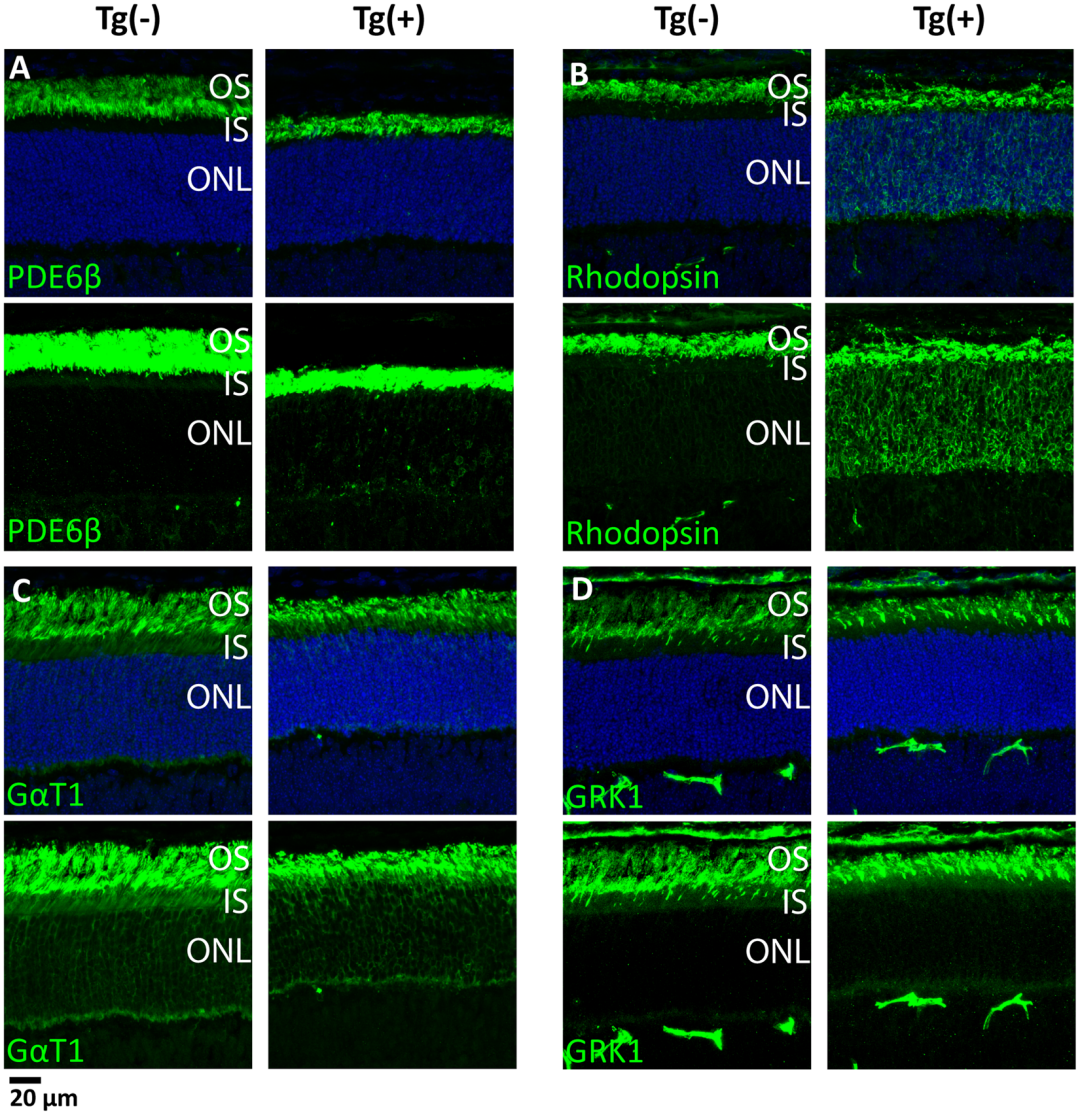
Defective localization of Rhodopsin in ARL2-Q70L animals. Immunofluorescence analysis of OS proteins in Tg(−) and Tg(+) samples at P13. PDE6β (**A**), Rhodopsin (**B**), GαT1 (**C**), and GRK1 (**D**) with unadjusted image intensity (Top) and enhanced image intensity to visualize immunoreactivity of lower abundance proteins in the ONL (Bottom). (Scale Bar = 20 μm).

The ARL2 homolog, ARL3, regulates trafficking of prenylated proteins to the rod OS^11,12^. Transgenic animals expressing ARL3-Q70L showed disrupted trafficking of prenylated PDE6 and normal distribution of rhodopsin^11^. Therefore, we wanted to determine if ARL2 serves a redundant function to ARL3 in this process. To answer these questions, we examined localization of rod OS-resident proteins using immunofluorescence microscopy at P13 (Fig. 5). Rhodopsin was dramatically mislocalized to the ONL surrounding the nuclei as well as the IS and synapse at P13 in ARL2-Q70L animals (Fig. 5, Panel B). Immunoreactivity for PDE6 was also intermittently found in the IS and ONL, but to a much lesser extent (Fig. 5, Panel A). The localization of rod transducin and GRK1 appears normal (Fig. 5, Panel C and D) This is contrary to the mislocalization seen in our previous work with the ARL3-Q70L model in which prenylated proteins GRK1, PDE6, and rod transducin were mislocalized to vacuole-like structures in the rod IS^11^. This observation adds support for a role of ARL2 in photoreceptor development and function that is different from its close paralogue ARL3.

### Abnormal elaboration of photoreceptor OS in ARL2-Q70L Mice

**Figure 6:**
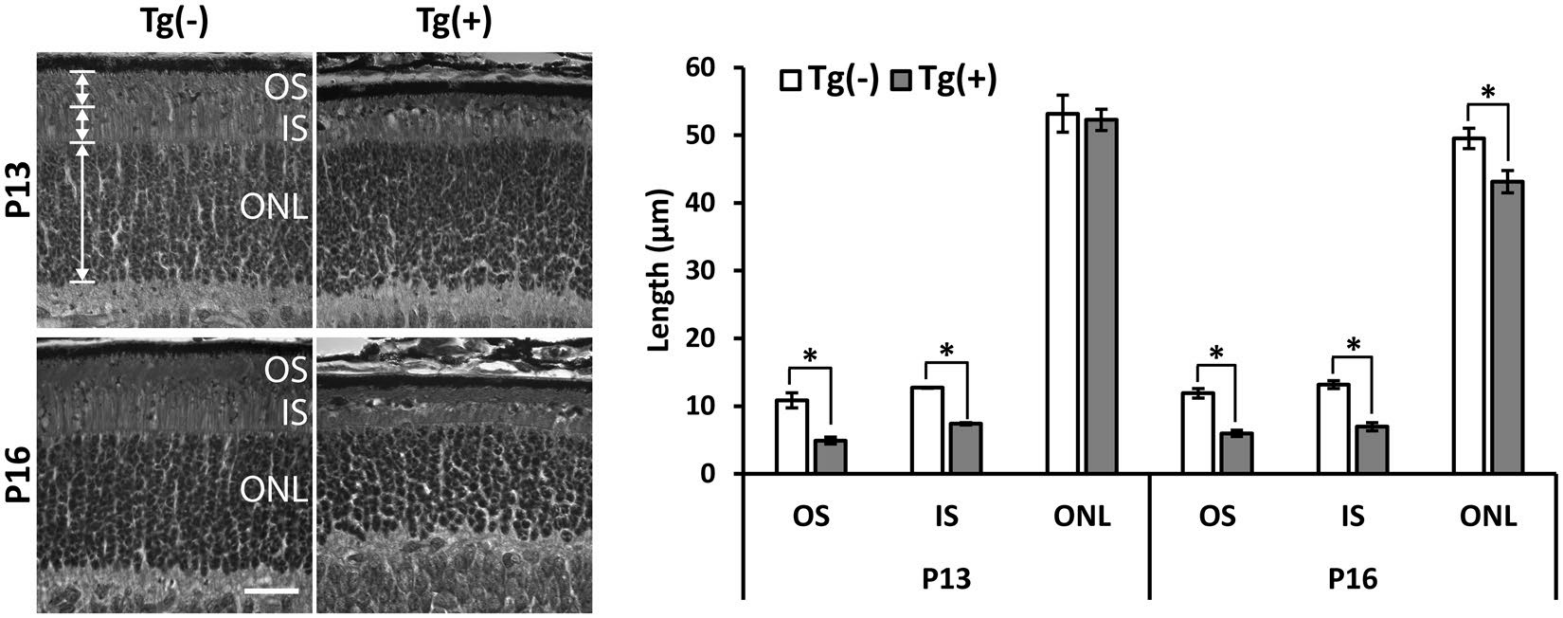
Aberrant elaboration of photoreceptor OS in ARL2-Q70L Mice. Representative toluidine blue stained retinal cross sections from Tg(−) and Tg(+) littermates at P13 and P16. Quantification of the OS, IS and ONL length from these animals at respective ages. n = 4, *p < 0.05. (scale bar = 20 μm).

The broad decrease in OS protein levels in the ARL2-Q70L transgenic line with no major loss in photoreceptor cells at P13, can be explained by a structural defect of the OS. For initial insight into photoreceptor cell structure, we examined the morphology of the toluidine blue stained retinal sections by light microscopy. We examined retinal sections from animals at P13 and P16, before ONL degeneration and after initial significant photoreceptor loss, respectively. Transgenic ARL2-Q70L had a significant ~2-fold decrease in the OS length at P13 (Fig. 6; P13: Tg(−) = 10.9 ± 1.1 μm versus Tg(+) = 4.9 ± 0.5, n = 4, p < 0.05). Furthermore, there was a significant ~1.7-fold decrease in IS length in Tg(+) at P13 (Fig. 6; P13: Tg(−) = 12.7 ± 0.1 μm versus Tg(+) = 7.4 ± 0.2 μm, n = 4, p < 0.001). However, there were no significant differences in ONL length between transgenic negative and positive at P13 (Fig. 6; P13: Tg(−) = 53.2 ± 2.7 μm versus Tg(+) = 52.3 ± 1.6 μm, n = 4, p = 0.79). Similar results were found at P16 except there was a minor, but significant reduction in the ONL length at this age. These data further support the conclusion that transgenic expression of ARL2-Q70L impedes the development of the photoreceptor outer segment.

### ARL2-Q70L expression results in defects in cilia regulation and OS development in rod photoreceptor cells

**Figure 7:**
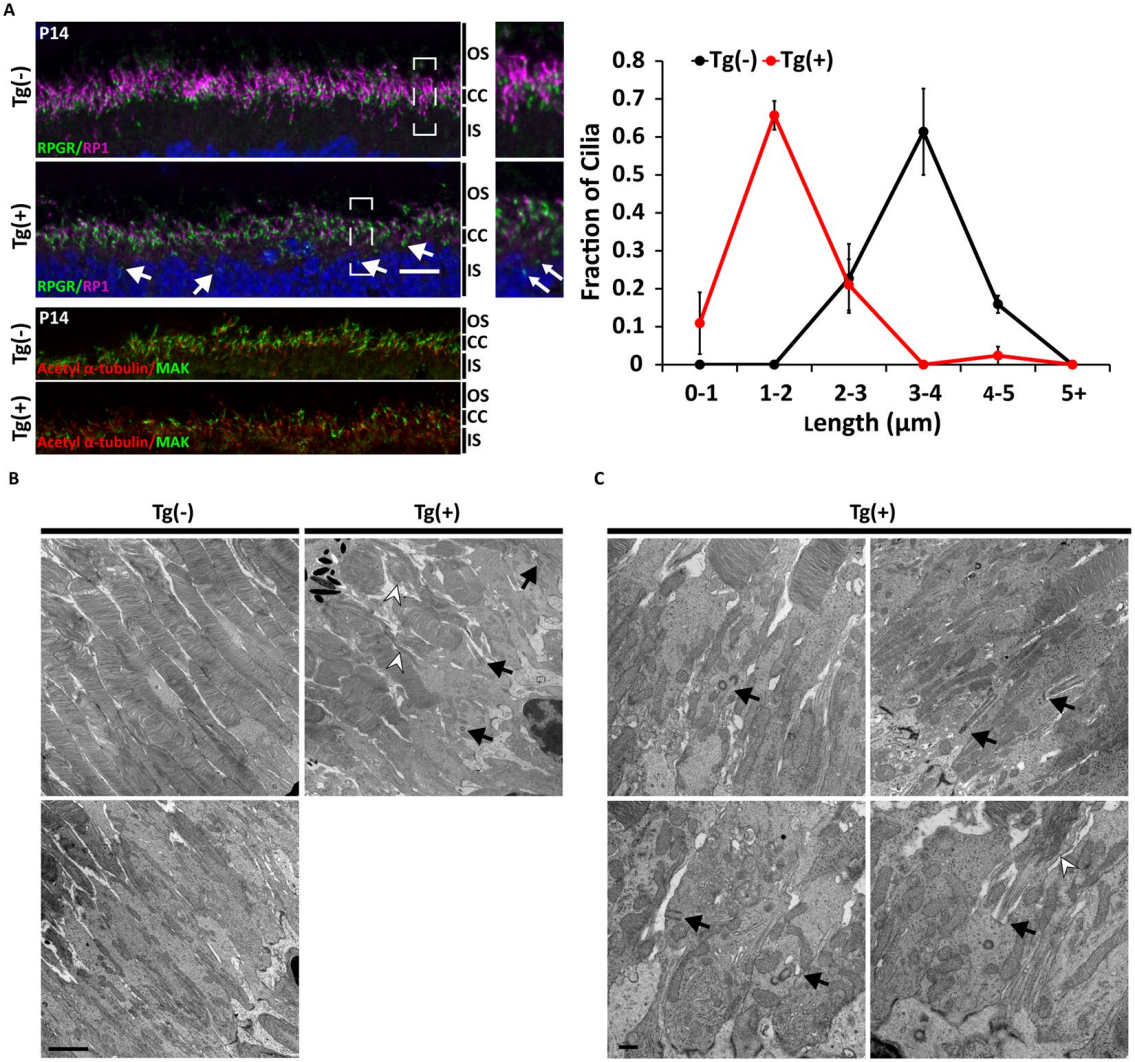
Defective development of photoreceptor axoneme and OS in rods expressing ARL2-Q70L. (**A**) Immunofluorescence analysis of retinal cross sections of Tg(−) and Tg(+) littermates at P14 showing immunoreactivity for DAPI (blue), acetyl α-tubulin (red), RPGR or MAK (green), and RP1 (magenta) (Scale Bar = 10 μm). A frequency distribution illustrating the fraction of total measured cilia (length from base of RPGR to tip of RP1 immunoreactivity) ranging between 0-1, 1-2, 2-3, 3-4, 4-5, and 5 + microns (White arrows indicate mislocalized cilia). n = 3. (**B**) Electron micrographs of retinal cross sections at low magnification of Tg(−) and Tg(+) littermates at P13 illustrating the structural abnormalities including misoriented, dysmorphic and shortened OS discs (white arrowheads), shorter IS length, and aberrantly localized cilia near the OLM (black arrows) in Tg(+) (Scale Bar = 2 μm). (**C**) High magnification electron micrographs highlighting ultrastructural defects. Black arrows indicate misoriented or mislocalized basal bodies and cilia. White arrowheads indicate misoriented/dysmorphic OS discs (Scale Bar = 0.5 μm).

ARL2 has recently been implicated in the regulation of ciliary length *in vitro*^7^. To explore this potential role in intact photoreceptor cells, immunofluorescence and transmission electron microscopy were performed to examine axonemal integrity and rod photoreceptor cell ultrastructure. RP1 and RPGR immunoreactivity were used to determine the length of the connecting cilium and axoneme in the ARL2-Q70L retina at P14 (Fig. 7A). Immunoreactivity of these two markers in control Tg(−) retina produced uniform staining at the IS-OS border with 1 to 2 μm connecting cilium extending to a 2–4 μm axoneme (Fig. 7A; Top Panel). However, in transgenic animals (Tg(+)) the IS-OS border is less apparent with a number of instances of cilium staining within the IS region near the outer limiting membrane (OLM) (Fig. 7A; Bottom Panel; White Arrows). Although the length of the connecting cilium, as measured by RPGR immunoreactivity is comparable to wild type tissue, the length of the axoneme (measured by RP1 immunoreactivity) is dramatically shorter (Fig. 7A; Bottom Panel). More specifically, ciliary length in Tg(+) animals was on average ~2-fold shorter than littermate controls with approximately 70% of cilia between 1 and 2 μm compared to the same proportion between 3 and 4 μm (Fig. 7A; Right). This reduction in axoneme length was also confirmed using anti-MAK antibodies which are used as a marker for the connecting cilium and axoneme (Fig. 7A; Bottom-Left).

To explore possible ultrastructural defects, electron microscopy was performed at P13 prior to the onset of photoreceptor cell death. Photoreceptor OSs were frequently shorter and misoriented in comparison to wildtype controls (Fig. 7B,C; White Arrowheads). Additionally, aberrantly localized cilia were found protruding from the lower portions of the IS near the OLM (Black Arrows) in the retina from transgenic positive animals while other cilia were localized normally at the apical IS. In contrast, there is a clear demarcation between the inner and outer segment in the transgenic negative littermates with all cilia properly localized at the apical IS (Fig. 7B,C). Similar to our findings from light microscopy, the IS was dramatically shorter. Overall, these results suggest that expression of ARL2-Q70L interferes with the development of the ciliary axoneme and the photoreceptor OS.

### ARL2-Q70L expression negatively affects ciliary stability in RPE1-hTERT cell lines

**Figure 8:**
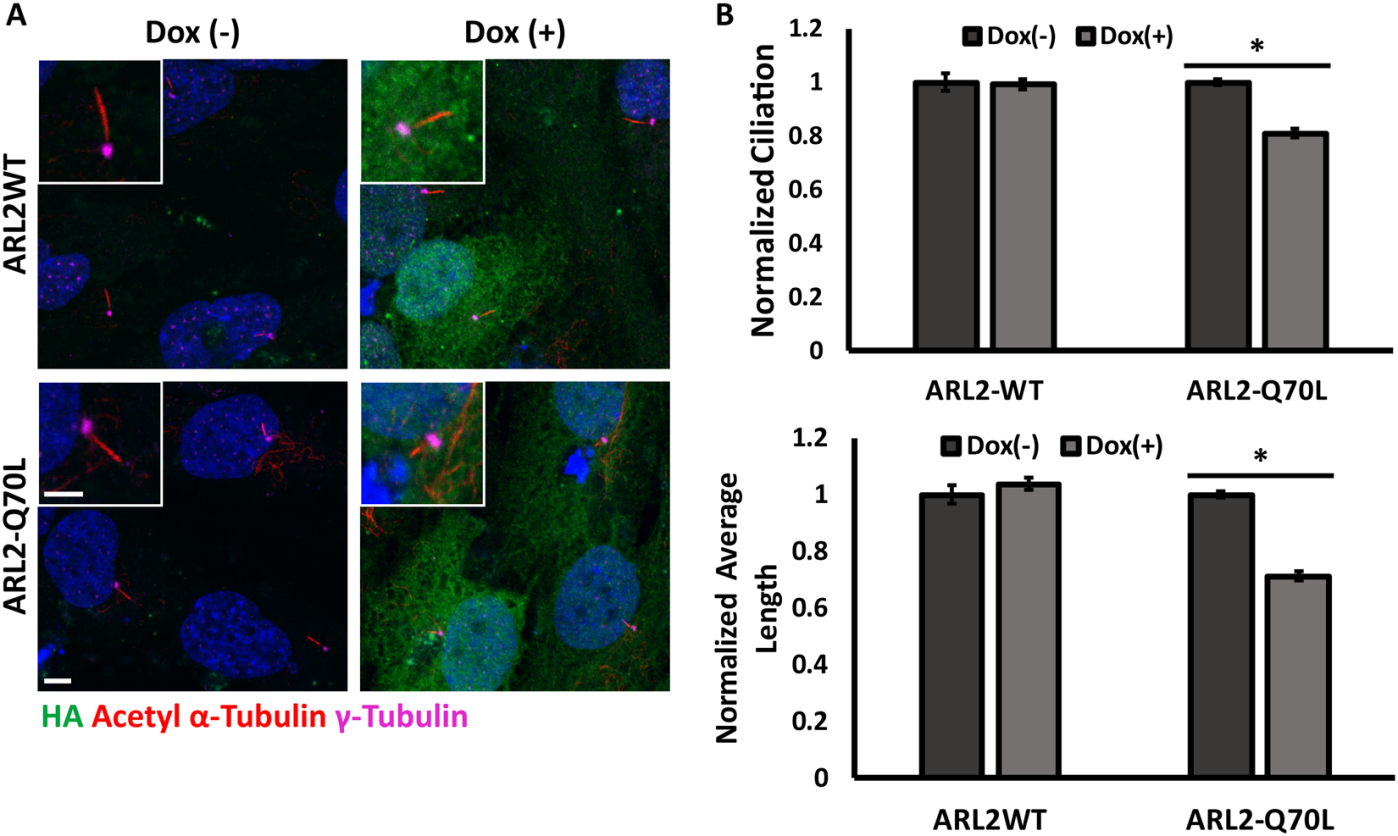
ARL2-Q70L expression affects ciliary stability and ciliary length in RPE1-hTERT cell lines. (**A**) Immunofluorescence analysis of RPE1-hTERT cell sublines with HA-tagged ARL2-WT or ARL2-Q70L at 48 hours of FBS(−) media incubation without (Dox(−)) or with (Dox(+)) doxycycline added at 24 hours of FBS(−) media incubation. Demonstrating immunoreactivity for DAPI (blue), HA (green), Acetyl α-tubulin (red), and γ-tubulin (magenta) (Scale Bar = 5 μm; Scale Bar (zoomed) = 3 μm). (**B**) Bar graphs illustrating the fraction ciliated (Ciliation) (Top) and average ciliary length (Bottom) normalized to Dox(−) control using at least 100 cells and 80 cilia per replicate. n = 3, *p < 0.05.

To determine if the decrease in axoneme length is specific to photoreceptor cells we generated stable RPE1-hTERT cell lines with a dox-inducible promoter driving expression of ARL2-WT or ARL2-Q70L. As described previously^23^, ciliation was induced by switching to serum free media. Doxycycline was added after 24 hr of serum starvation to the medium to induce expression of ARL2 protein from the lentiviral vector. Induction of transgene expression 24 hr after serum starvation was completed to isolate the effects of the transgenic protein on cilia stability based on previous work demonstrating that the number of cilia remain unchanged from 24 hr to 48 hr of serum starvation^23^. Immunoreactivity to γ-tubulin and acetylated α-tubulin was used to locate and measure the length of cilia in these cells. We observed 20% reduction in the fraction of ciliated cells with a 30% decrease in the length of cilia in ARL2-Q70L expressing cells compared to un-induced (Dox(−)) controls or to cells expressing wild type ARL2 protein (Fig. 8A,B). Additionally, expression of wild type ARL3 or dominant active ARL3-Q71L did not affect cilia length of the rate of ciliation (Cilia length: ARL3-WT – Dox(−) 2.49 ± 0.02 μm vs. Dox(+) 2.42 ± 0.05 μm, p = 0.36; ARL3-Q71L – Dox(−) 2.43 ± 0.02 μm vs. Dox(+) 2.65 ± 0.10 μm, p = 0.17; Fraction of ciliated cells/total cells: ARL3-WT – Dox(−) 0.59 ± 0.02 vs. Dox(+) 0.57 ± 0.06, p = 0.75; ARL3-Q71L – Dox(−) 0.65 ± 0.07 vs. Dox(+) 0.53 ± 0.03, p = 0.25; n = 3 for all endpoints). These data confirm the results obtained in the ARL2-Q70L transgenic model and further support the role for ARL2 in ciliary stability and maintenance of axoneme length.

## Discussion

In this study, we have discovered a novel regulator of rod photoreceptor development and function, ARL2. Our first indication that ARL2 plays a role in OS morphogenesis was the endogenous mRNA expression profile, which demonstrates a “switch”-like spike in retinal message levels at approximately P9, a critical period of OS elaboration (Fig. 1A). Furthermore, we observed localization of endogenous ARL2 in the area of the connecting cilium via immunofluorescence microscopy. In order to study ARL2 in rod photoreceptor neurons, we generated a dominant active transgenic mouse model that expresses ARL2-Q70L mutant under a rod promoter (Rhop). The expression of the transgenic protein was 40% the level of endogenous ARL2 and the total amount of ARL2 (transgenic + endogenous) in transgenic animals only 14% greater than the total ARL2 protein in transgenic negative animals. This result suggests a strong mechanism of regulation of ARL2 protein levels and the downregulation of native protein in our model to compensate for the expression of transgenic ARL2-Q70L. Interestingly, this finding is unique to expression of ARL2-Q70L as it was not present in our previous work with transgenic expression of ARL3-Q71L, which showed as much as 300% more transgenic ARL3-Q71L compared to controls^11^. This strengthens the idea that the phenotypes presented here do not result solely from effects of overexpression of exogenous protein. With this model, we demonstrate that expression of ARL2-Q70L results in early defects in OS development followed by slow photoreceptor degeneration. Additional support for the role for ARL2 in OS morphogenesis comes from recently described animal model lacking ARL2 in the retina^24^.

The phenotype observed in ARL2-Q70L model is distinct from that of the ARL3-Q71L lines generated for previous work^11^. Specifically, ARL3-Q71L transgenic animals exhibit normal photoreceptor elaboration and function at P20 with a rapid decline in function between the ages of P30 and P70 and concomitant degeneration of rods by P70. ARL2-Q70L transgenic animals have a decrease in the length of the photoreceptor OS and IS as early as P13 and abnormal rod function at P16 with a slow decline and degeneration up to P100. Interestingly, the localization of ARL2 and ARL3 are different in photoreceptor cells. While native ARL2 and ARL2-Q70L is confined to the IS, native ARL3 and ARL3-Q71L is present in both IS and OS^11^. Previous studies suggest that ARL2 may act similarly to ARL3 as a selective regulator for prenylated protein trafficking^9–11^. However, we observed minor mislocalization of the majority of prenylated proteins to the OS, which we attribute to a stunted OS. This is in contrast to ARL3-Q71L transgenic animals, which show progressive mislocalization of PDE6 and other prenylated proteins with no effect on OS elaboration. Immunoblotting from ARL2-Q70L retina shows a uniform decrease in the levels of OS proteins and not a selective decrease in prenylated proteins as occurs in ARL3-Q71L transgenic retina. Although we cannot completely rule out that ARL2 may act in prenylated protein trafficking, we can say that it does not appear to be its primary role *in vivo*. Unlike ARL2-Q70L, neither ARL3 wildtype or dominant active ARL3-Q71L expression in RPE1-hTERT cells resulted in abnormalities in ciliation or axonemal length. These data demonstrate a novel role for ARL2 in rod photoreceptor cells that is distinct from its homolog ARL3.

We believe the mislocalization of rhodopsin in ARL2-Q70L animals is a consequence of the morphological defects in the OS elaboration, similar to other cases where shortening of the OS or loss of the connecting cilium results in mislocalization of rhodopsin^12,25^. However, it is important to note that knockout and heterozygote mice for rhodopsin result in the absence of or shorter OS, respectively^26–28^. Additionally, mistrafficking of rhodopsin due to transgenic expression of a rhodopsin Pro23His mutant results in abnormal development of the OS and destabilization of OS discs^29,30^. While we cannot completely rule out that ARL2 disrupts trafficking of rhodopsin thereby affecting OS elaboration, it should be noted that the phenotype of the ARL2-Q70L transgenes is different from the rhodopsin knockout in two key aspects: the rhodopsin knockout does not produce mislocalization of the primary cilia or shortening of the inner segment^27^. Regardless of the mechanism leading to rhodopsin mislocalization, it is likely that the slow degeneration of photoreceptors in ARL2-Q70L animals results from accumulation and overload of degradation pathways by rhodopsin^28,31–33^.

We demonstrate that expression of ARL2-Q70L leads to decreased stability of cilia and length of the ciliary axoneme both in the specialized primary cilium of the photoreceptor OS and the primary cilium of RPE1-hTERT. ARL2BP, a previously identified interacting partner of ARL2, was recently shown to be involved in the development of cilia in photoreceptor cells in a mouse model^34^. As previously shown, mutations in ARL2BP results in retinitis pigmentosa and one specific mutation, c.134 T > G (p.Met45Arg), disrupts binding to ARL2^7^. Furthermore, knockdown of either ARL2 or ARL2BP results in shortening of cilia in RPE cell lines^7^. Notably, the active ARL2-GTP (and not ARL2-GDP) selectively binds to ARL2BP. Therefore, it is expected that ARL2-Q70L would sequester ARL2BP preventing its proper function and resulting in stunted cilia *in vivo*. Although these data suggest a compelling role for ARL2BP in our model, our current studies do not support the interaction of ARL2 with ARL2BP in photoreceptor cells^34^.

In addition, Newman *et al*. (2014) reported that ARL2 serves a role in mitochondrial morphology, motility, and maintenance of ATP levels that is independent of its role in tubulin regulation. However, only knockdown and expression of the dominant inactive mutant (ARL2-T30N) had effects on mitochondria while expression of the dominant active mutant (ARL2-Q70L) had no effects on mitochondrial endpoints and the greatest effect of all treatment groups on loss of microtubules^15^. Although we did not directly measure mitochondrial function and morphology, mitochondria appeared grossly normal and comparable to controls in ultrastructural analysis of ARL2-Q70L retina.

The photoreceptor cilium is a microtubule based-organelle containing the basal body, connecting cilium, and the axoneme, which extends into the OS. This organelle is the foundation of the photoreceptor OS and therefore necessary for the specialized function of capturing light. Formation of the cilium is complex and depends on the polymerization of tubulin to form microtubules. Interestingly, ARL2 is a known regulator of microtubules and tubulin in cell culture systems and expression of ARL2-Q70L in multiple cell lines results in destruction of microtubules and cell cycle arrest^14,20^. In addition, expression of wildtype ARL2 rescues microtubules from tubulin binding cofactor D (TBCD)-mediated destruction^20^. A similar effect on microtubule stability was observed in the adenocarcinoma cell line, MCF7, where lines with the greatest expression of ARL2 showed higher content of polymerizable tubulin heterodimers and low expressing lines had reduced content^35^. ARL2 was recently implicated in the regulation of soluble polymerizable tubulin pools, a role that relies on the formation of a “TBC-DEG” chaperone complex composed of ARL2 and the tubulin chaperones TBCD and tubulin binding cofactor E (TBCE)^19^. Furthermore, the ARL2-TBCD interaction has been found to be critical for maintenance of microtubule densities in cells and the TBCD-ARL2-β-tubulin trimer is fundamental to microtubule dynamics^36^. Considering the major structural defects associated with ARL2-Q70L expression, including dysmorphic OS and IS, it is tempting to suggest that ARL2 plays a role in regulation of the polymerizable and or maintenance of the pool of tubulin in photoreceptors. Importantly, ARL2BP does not seem to play a role in tubulin regulation, at least outside of its minor effects on ciliary length^14,15^. Based on our findings and previous studies, future work will delve deeper into the role of ARL2 in coordination with “TBC-DEG” and the more fundamental TBCD-ARL2-β-tubulin trimer to answer the question of how this dysregulation leads to OS and IS dysmorphogenesis.

Finally, we demonstrate that ARL2-Q70L expression results in aberrant localization of the photoreceptor cilium. Some of the cases of this phenotype can be explained by impending photoreceptor cell death that results in retraction of the OS and IS toward the cell body. However, there were many cases where the basal body and axoneme were localized at the base of the IS protruding from the cell in different directions. Because the transgene is expressed after the point of apical localization of the basal body prior to axoneme elongation, it is likely that there is a defect in the process that positions this organelle at the apical surface, allowing the IS and OS to grow distally. We speculate that axonemal growth is required for proper OS development and that the cells containing mislocalized cilia may express the transgene slightly earlier such that they are unable to produce even a rudimentary OS.

Current literature suggests that expression of ARL2-Q70L results in decrease of soluble tubulin pools, likely through interfering with tubulin folding. We posit that reduced pools of polymerizable tubulin or disruption of the TBCD-ARL2-β-tubulin trimer by constitutively active ARL2-Q70L leads to defects in ciliary development. Our results show dramatic effects on the architecture of photoreceptor cells, including the spatial organization of the cilia, basal body, and ciliary associated centriole. This disruption likely results in impaired development of the rod OS and progressive cell death. Future studies are necessary to fully elucidate the mechanisms underlying ARL2 regulation of tubulin, particularly at the connecting cilium of photoreceptor cells where the ARL2 protein is concentrated.

## Methods

### RNA isolation and qRT-PCR from retina

Mouse eyes were enucleated at indicated time points and dissected under microscope (Zeiss Stemi DV4) to isolate the retina. RNA was isolated with TRI reagent (Sigma) according to manufacturer’s guidelines. Reverse-transcription PCR reactions containing 0.1–0.5 μg RNA were primed with Oligo-dT and random hexamers to generate cDNA. Expression of *Arl2* and *Arl3* was quantified by SYBR-green qPCR normalized to the expression of Glyceraldehyde-3-Phoshpate Dehydrogenase (Gapdh). qRT-PCR primers for genes assessed were: *Gapdh* – Forward = AGACGGCCGCATCTTCTT and Reverse = TTCACACCGACCTTCACCAT, *Arl3* – Forward = TACTCCTGGGCTTGGACAAC and Reverse = TGTGACTGCACGCTTTTGAT, *Arl2* – Forward = GAGCACCGCGGATTCAA and Reverse = GCAAAGATGAGGAGGGTTCG.

### ARL2-Q70L transgenic mouse model generation

Transgenic model generation followed that of previously published work^11^. Briefly, a full-length human *ARL2* clone (Thermo Scientific) was used to generate an *ARL2* mutant construct with a tandem C-terminal hemagglutinin (HA) and FLAG tag that is driven by a 4.4 kb rhodopsin promoter (Rhop). Of note, human ARL2 is 96% identical to the mouse protein and carries only one non-conservative amino acid substitution, R153 > C. This *ARL2* construct was modified by site-directed mutagenesis to produce a glutamine (Q) to leucine (L) variant at position 70 (Q70L) using a mutant oligonucleotide (5′-CCG CAG GGA CTT CAG GCC ACC CAC ATC-3′). After amplification, the PCR product was modified with a tandem C-terminal HA and FLAG tag and then cloned behind a 4.4 kb rhodopsin promoter. All clones selected were sequenced in both directions to confirm that there are no unintended mutations. After removal from the plasmid by digestion, *Rhop-ARL2-Q70L-polyA* was purified by agarose gel electrophoresis and elution. This purified DNA fragment was injected into pronuclei of oocytes from superovulated FVB/N females (WVU Transgenic Core Facilities) and implanted in to pseudo-pregnant CD-1 females. Over 8 founder lines were examined and the lines utilized in this publication were selected because they exhibited the highest homogeneity of transgene expression in photoreceptor cells.

### Genotyping and Founder Line Maintenance

ARL2-Q70L transgenic founders were initially identified by PCR of genomic DNA isolated from tail snips or ear punches using the following primers: 5′-GGA TCG TGA ATC AGC CTC TGG CTT-3′ and 5′-CTG CAT GCG CTG GCG GTC TGC-3′. PCR reactions utilized NEB quick load Taq polymerase with the following conditions: 1. 95 °C for 2 minutes, 2. 95 °C for 30 seconds, 3. 59 °C for 30 seconds, 4. 72 °C for 45 seconds, and 5. 72 °C for 5 minutes with steps 2–4 repeated for 33 cycles. Identified founders were crossed with 129/SV-E mice (Charles River) over multiple generations to eliminate the *Pde6b*^*rd1*^ mutation present in FVB mice (verified by PCR genotyping)^37^ as well as to ensure transgenic expression was resulting from a single locus. Animals were maintained under a 12-hour light/12-hour dark cycles with food and water *ad libitium*. All procedures were conducted in accordance with the NIH Guide for the Care and Use of Laboratory Animals. All proposed experiments were reviewed and approved by the Institutional Animal Care and Use Committee of West Virginia University.

### Electroretinographic (ERG) analysis

Electroretinography (ERG) was performed as previously described using UTAS Visual Diagnostic System with BigShot Ganzfeld with UBA-4200 amplifier and interface, and EMWIN 9.0.0 software (LKC Technologies, Gaithersburg, MD, USA)^11^. After dark-adaptation overnight, a 1:1 mixture of tropicamide:phenylephrine hydrochloride was used to dilate the test animal’s eyes. Mice were placed on a heated platform with continuous flow of 1.5% isofluorane with 2.5 liters per minute oxygen flow rate and a subcutaneous reference electrode. Corneal electrodes were placed making contact with the cornea using hypromellose solution (2% hypermellose in PBS) (Gonioscopic Prism Solution, Wilson Ophthalmic, Mustang, OK, USA). Scotopic responses were measured by exposure to flashes of white LED light starting at low intensities and proceeding to higher intensities. Afterward, mice were light-adapted for 10 minutes under 30 cd/m^2^ rod-saturating white background light for phototopic response measurements.

### Generation of Antibody against full-length ARL2

ARL2 antibody was generated as previously described^11^. Briefly, ARL2 with a C-terminal his-tag was expressed in Origami *E*. *coli* strain (Novagen). Origami cells were grown to an OD_600_ ≈ 0.6 and protein production was induced with 1 mM IPTG for 18 hours at 18 °C. The protein was purified from the soluble fraction using a Nickel His Affinity Column and the eluate was supplied to Pacific Immunology Corp. for generation of the antibody in rabbits. Antibody serum was then purified against a GST-ARL2 fusion protein and tested via western blot and immunofluorescence microscopy in both HEK 293 cells expressing ARL2 and retinal extracts.

### Immunoblots

Flash frozen dissected retinal samples from enucleated eyes were sonicated in phosphate buffered saline (PBS) (137 mM NaCl, 2.7 mM KCl, 4.3 mM Na_2_HPO_4_·7H_2_O, 1.4 mM KH_2_PO_4_, with protease inhibitor cocktail (Roche)). After measurement of protein concentration using spectrophotometry (NanoDrop - Thermo Fisher Scientific, Inc), a sample volume equal to 150 µg total protein was loaded per well of a 12% or 15% polyacrylamide SDS-PAGE resolving gel (Criterion Midi format, Bio-Rad). Proteins were then resolved and transferred onto polyvinylidene difluoride membranes (Immunobilon-FL, Millipore, Billerica). Membranes were blocked for 30 minutes at room temperature (Western Blot Blocking Buffer – Rockland Inc.) and incubated with primary antibodies at a dilution of 1:2000 overnight at 4 °C. After washing in PBST (PBS with 0.1% Tween-20) 3 times for 5 minutes, secondary antibodies (goat anti-rabbit Alexa 680 (or 800), goat anti-rat Alexa 800, or goat anti-mouse Alexa 680 (Invitrogen)) were applied for 30 minutes at room temperature. After washing again in PBST, blots were imaged using the Odyssey Infrared Imaging System (LI-COR Biosciences, Lincoln, NE, USA).

### Antibodies

The following antibodies were used for immunoblot and immunofluorescence analysis at a dilution of 1:2000 or 1:1000, respectively, unless otherwise noted: rabbit anti-PDE6α (Pierce), rabbit anti-PDE6β (Pierce), rabbit anti-PDE6γ (Pierce), assembled PDE6 (ROS1) (generously provided by Drs. Ted Wensel (Baylor College) and Rick Cote (University of New Hampshire), rabbit anti-Transducin-αt1 (Santa Cruz), mouse anti-cytochrome c oxidase subunit I (COX I) (MS404 MitoSciences), rabbit anti-Transducin-γt1 (Santa Cruz), mouse anti-GRK1 (Thermo Fisher), mouse anti-CNGA1 (UC Davis/NIH NeuroMab Facility), rabbit anti-RDS-c (Peripherin-2) (Gabriel Travis, University of California, Los Angeles, CA), mouse anti-1D4 (rhodopsin) (gift from Dr. Ted Wensel, Baylor Collect of Medicine), rat anti-HA antibody (Roche), chicken anti-RP1 (gift from Dr. Qin Liu Mass. Eye and Ear, Boston, MA), and rabbit anti-ARL2 (1:1000) (see above). DAPI (1:1000) (4′,6-diamindino-2-phenylindole, Invitrogen) and propidium iodide (PI) (1:2000) (EMD Millipore, Billerica, MA, USA) were also used in indirect immunofluorescence.

### Immunofluorescence Analysis

Indirect immunofluorescence was utilized to analyze protein localization in retinal cross-sections. For general immunofluorescence studies, enucleated eyes were immersed in 4% PFA in PBS for 15 minutes, after which the anterior segments were removed and the eyecups were placed back in fixative for a total of 3 hours. Fixed eyecups were washed with PBS and kept in 20% sucrose in PBS overnight at 4 °C. After incubation in a 1:1 mixture of 20% sucrose solution:OCT (Cryo Optimal Cutting Temperature Compound, Sakura) for 2 hours, eyecups were flash frozen in OCT. Alternatively, for staining of ciliary markers, anterior segments were dissected immediately after enucleation and eyes were placed in 4% PFA for 30 seconds. These eyecups were flash frozen in OCT. Serial retinal cross-sections at 16 µm thickness (or 10 μm for ciliary markers) were mounted on Superfrost Plus slides (Fisher Scientific). Sections were blocked for 1 hour (PBS with 5% goat serum, 0.5% Triton X-100, 0.05% sodium azide) and incubated with primary antibody overnight at 4 °C then washed with PBS + 0.1% Triton X-100 three times for 5 minutes. Secondary antibodies (DAPI nuclear stain 405, anti-Rat 568, anti-Rabbit 488 (or 568), anti-mouse 488 (or 568)) were incubated for 1 hour at room temperature at a dilution of 1:1000. Sections were washed again and cover slipped with ProLong Gold (Life Technologies). Confocal imaging was performed using the Nikon C2 Confocal Microscope System. Images were processed using ImageJ image processing software.

### RPE1-hTERT Stable Cell Line Generation and Analysis

RPE1-hTERT cells were cultured according to suppliers recommendations from ATCC (https://www.atcc.org). For stable cell subline generation ARL2-HA, ARL2-Q70L-HA, Arl3-HA and Arl3-Q70L-HA were subcloned into pLUT backbone under doxycycline inducible promoter. Viral particles were generated as previously described^23^. Infections were followed by selection with 8 µg/ml of puromycin until stably growing clones were established. To induce ciliation, stable cells lines from above were incubated in the absence of FBS (FBS(−) for 48 hours. After 24 hours of FBS(−) incubation, doxycycline was added to induce expression of ARL2-HA, ARL2-Q70L-HA, Arl3-HA or Arl3-Q70L-HA. Measurements of ciliation (fraction of cells expressing the exogenous protein that have cilia) and cilia length were completed 48 hours after FBS(−) incubation using immunofluorescence analysis with acetyl-α-tubulin (axonemal marker) co-labeled with γ-tubulin (basal body marker). Measurements were compared between dox(−) controls and dox(+) samples.

### Toluidine Blue Histological Analysis

Eyes were enucleated with the 12 o’clock position marked for reference with a red lipophilic dye and immediately fixed in Excalibur Pathology Fixation Buffer at room temperature. Fixed eyes were embedded in paraffin, sectioned at 2 μm, and stained with toluidine blue by Excalibur Pathology (Norman, OK). Images were captured with a Nikon light microscope. Analysis, including measurement of OS, IS, and ONL length was performed using ImageJ.

### Ultrastructural Analysis

Enucleated eyes fixed in 2% paraformaldehyde, 2.5% glutaraldehyde, 0.1 M cacodylate buffer, pH 7.5 for 30 minutes at room temperature were dissected removing the anterior segment and lens then fixed further for at least 2 days. Fixed eyecup was dissected into 6–8 wedge-shaped pieces. Wedges were dehydrated in a graded ethanol series, osmicated, en bloc stained with uranyl acetate, and then embedded in Polybed 812 (PolySciences, Inc., Warrington, PA, USA). Semi-thin (1 μm) sections were collected onto glass slides, stained with toluidine blue, and visualized using a Zeiss Axioimager 2 microscope equipped with EC Plan-Neofluar 40 × (N.A. 0.75) and 100 × (1.3 N.A.) objectives. Thin sections (ca. 80 nm) from selected wedges were collected onto copper grids, stained with 2% uranyl acetate and lead citrate, and imaged using an FEI Morgagni transmission electron microscope at 80 kV.

### Experimental design and Statistical Analysis

All quantitative analysis was performed on age-matched littermate wild-type controls and ARL2-Q70L transgenic mice with a minimum sample size of 3 animals per group compared. For immunohistochemical analysis, at least 4 sections were imaged per sample and data were derived from at minimum *n* > 3 independent experiments. Data are presented as mean ± standard error. Unpaired Student’s t tests were conducted to compare measured values between control and mutant samples. For cilia measurements, 80–100 cilia were measured for each animal or culture (*n* = 3). Image and densitometry analysis were performed using ImageJ-FIJI 1.50i along with the Bio-Formats plugin (NIH).

## Data Availability

The datasets generated during and/or analyzed during the current study are available from the corresponding author upon request.
